# Improvement in the survival rates of extracorporeal membrane oxygenation-supported respiratory failure patients: a multicenter retrospective study in Korean patients

**DOI:** 10.1186/s13054-018-2293-5

**Published:** 2019-01-03

**Authors:** Moon Seong Baek, Sang-Min Lee, Chi Ryang Chung, Woo Hyun Cho, Young-Jae Cho, Sunghoon Park, So-My Koo, Jae-Seung Jung, Seung Yong Park, Youjin Chang, Byung Ju Kang, Jung-Hyun Kim, Jin Young Oh, So Hee Park, Jung-Wan Yoo, Yun Su Sim, Sang-Bum Hong

**Affiliations:** 1 Department of Pulmonary and Critical Care Medicine, Asan Medical Center, University of Ulsan College of Medicine, 88, Olympic-ro 43-gil, Songpa-gu, Seoul, 05505 Republic of Korea; 2 Division of Pulmonary and Critical Care Medicine, Department of Internal Medicine, Seoul National University College of Medicine, Seoul, Republic of Korea; 3 Department of Critical Care Medicine, Samsung Medical Center, Seoul, Republic of Korea; 4 Department of Internal Medicine, Pusan National University Yangsan Hospital, Yangsan-si, Gyeongsangnam-do Republic of Korea; 5 Division of Pulmonary and Critical Care Medicine, Department of Internal Medicine, Seoul National University Bundang Hospital, Seongnam-si, Gyeonggi-do Republic of Korea; 6 Division of Pulmonary and Critical Care Medicine, Department of Medicine, Hallym University Sacred Heart Hospital, Anyang-si, Gyeonggi-do Republic of Korea; 7 Division of Pulmonary and Allergy Medicine, Department of Internal Medicine, Soonchunhyang University Hospital, Seoul, Republic of Korea; 8 Department of Thoracic and Cardiovascular Surgery, Anam Hospital, Korea University College of Medicine, Seoul, Republic of Korea; 9 Department of Internal Medicine, Chonbuk National University Hospital, Jeonju-si, Jeollabuk-do Republic of Korea; 10 Division of Pulmonary and Critical Care Medicine, Department of Internal Medicine, Inje University College of Medicine, Sanggye Paik Hospital, Seoul, Republic of Korea; 11 Division of Pulmonology, Department of Internal Medicine, Ulsan University Hospital, Ulsan, Republic of Korea; 12 Division of Pulmonary and Critical Care Medicine, Department of Medicine, Bundang CHA Hospital, Seongnam-si, Gyeonggi-do Republic of Korea; 13 Division of Pulmonary and Critical Care Medicine, Department of Internal Medicine, Dongguk University, Ilsan Hospital, Goyang-si, Gyeonggi-do Republic of Korea; 14 Department of Pulmonary and Critical Care Medicine, Kyung Hee University Hospital, Gangdong, Seoul, Republic of Korea; 15 Department of Internal Medicine, College of Medicine, Gyeongsang National University Hospital, Jinju, Gyeonsangnam-do Republic of Korea; 16 Division of Pulmonary and Critical Care Medicine, Department of Medicine, Hallym University Kangnam Sacred Heart Hospital, Seoul, Republic of Korea

**Keywords:** Extracorporeal membrane oxygenation, Utilization, Survival

## Abstract

**Background:**

Although the utilization of extracorporeal membrane oxygenation (ECMO) is increasing and its technology is evolving, only a few epidemiologic reports have described the uses and outcomes of ECMO. The aim of this study was to investigate the changes in utilization and survival rate in patients supported with ECMO for severe respiratory failure in Korea.

**Methods:**

This was a multicenter study on consecutive patients who underwent ECMO across 16 hospitals in Korea. The records of all patients who required ECMO for acute respiratory failure between 2012 and 2015 were retrospectively reviewed, and the utilization of ECMO was analyzed over time.

**Results:**

During the study period, 5552 patients received ECMO in Korea as a whole, and a total of 2472 patients received ECMO at the participating 16 hospitals. We analyzed 487 (19.7%) patients who received ECMO for respiratory failure. The number of ECMO procedures provided for respiratory failure increased from 104 to 153 during the study period. The in-hospital survival rate increased from 30.8% to 35.9%. The use of prone positioning increased from 6.8% to 49.0% (*p* < 0.001), and the use of neuromuscular blockers also increased from 28.2% to 58.2% (*p* < 0.001). Multiple regression analysis showed that old age (OR 1.038 (95% CI 1.022, 1.054)), use of corticosteroid (OR 2.251 (95% CI 1.153, 4.397)), continuous renal replacement therapy (OR 2.196 (95% CI 1.135, 4.247)), driving pressure (OR 1.072 (95% CI 1.031, 1.114)), and prolonged ECMO duration (OR 1.020 (95% CI 1.003, 1.038)) were associated with increased odds of mortality.

**Conclusions:**

Utilization of ECMO and survival rates of patients who received ECMO for respiratory failure increased over time in Korea. The use of pre-ECMO prone positioning and neuromuscular blockers also increased during the same period.

## Background

Extracorporeal membrane oxygenation (ECMO), which provides respiratory and/or cardiac support, allows treatment of patients with refractory gas-exchange abnormalities [1]. The use of ECMO to support patients with respiratory failure is increasing worldwide following the use of ECMO for severe acute respiratory failure during the 2009 influenza A pandemic [2–5]. Recently, the EOLIA trial reported that in patients with severe acute respiratory distress syndrome (ARDS) there was no significant difference in 60-day mortality between patients who received early ECMO and those who received conventional mechanical ventilation that included ECMO as rescue therapy [6]. However, crossover to ECMO occurred in 28% of patients in the conventional group, who showed a high mortality rate of 57%. This suggests that ECMO can be used in severe ARDS patients who do not benefit from conventional treatment.

Survival of patients who received ECMO is also gradually increasing over time [7]. A recent epidemiologic report in Germany showed that ECMO utilization for severe respiratory failure significantly increased from 2007 until 2012, and in-hospital survival increased over time as well [8]. Sauer et al. [9] reported that the annual rates of ECMO cases increased by 433% from 2006 to 2011 in the United States, and that, albeit not statistically significant, there was an improving trend in the survival rate as well. In a single-center study in Korea, the survival rates associated with the ECMO procedure increased between 2009 and 2011 [10]. However, as we have previously reported, there was a discrepancy in the survival rate between those of the Extracorporeal Life Support Organization (ELSO) registry and Korean ECMO patients [11]. The in-hospital survival rate of ECMO-treated patients with acute respiratory failure was 46% from 2014 to 2015 in Korea, whereas the survival rate was 58% in the ELSO registry patients [7]. Also, we have suggested that age is an important factor in the survival of patients who received ECMO. Therefore, we sought to determine whether there has been an improvement in the survival rate of patients who received ECMO support for acute respiratory failure in Korea. Specifically, we evaluated the changes over time in the survival rates of patients supported with ECMO for severe respiratory failure and the factors associated with the survival rate.

## Methods

### Study design

This was a multicenter study of consecutive patients who received ECMO at 16 hospitals in Korea. The records of all patients who required ECMO for acute respiratory failure between 2012 and 2015 were retrospectively reviewed and the utilization of ECMO was analyzed over time. The decision to use ECMO was made at the discretion of the attending physicians at each center without standardization. The study protocol was approved by the institutional review board of Asan Medical Center, and by the local institutional review boards of all other participating centers. The requirement for informed consent was waived due to the retrospective design of the study.

### Data collection

Data were collected from electronic medical records of patients older than 19 years who received ECMO support. Included variables were as follows: demographic information, Acute Physiology and Chronic Health Evaluation (APACHE) II and Sequential Organ Failure Assessment (SOFA) scores at intensive care unit (ICU) admission, etiology of respiratory failure, cardiac arrest, immunocompromised status, central nervous system (CNS) dysfunction, pre-ECMO hemodynamic data, mechanical ventilation parameters, and arterial blood gas data. Immunocompromised status and CNS dysfunction were defined according to the RESP study [12]. Immunocompromised status included hematological malignancies, solid tumors, solid-organ transplantation, high-dose or long-term corticosteroid and/or immunosuppressant use, and human immunodeficiency virus infection. CNS dysfunction included diagnoses of neurotrauma, stroke, encephalopathy, cerebral embolism, seizure, and epileptic syndrome. We collected information on adjunctive therapy such as the use of vasopressors, steroids, continuous renal replacement therapy (CRRT), prone positioning, nitric oxide, bicarbonate infusion, and neuromuscular blockers. We also collected data such as the ECMO mode, ECMO duration, duration of mechanical ventilation to ECMO initiation, hospital stay, and tracheotomy. The ECMO mode was categorized as veno-venous, veno-arterial, and veno-arteriovenous. Outcome variables of the study were survival at discharge and ECMO weaning (survival within 48 h after weaning from ECMO).

### Statistical analysis

Demographics, pre-ECMO parameters, and outcomes were compared between 2012 and 2015. Differences with *p* < 0.05 were considered statistically significant. Categorical variables are expressed as the number (percentage). Continuous variables are expressed as the median (interquartile range). Pearson’s chi-square test or Fisher’s exact test was used to compare categorical data. The Kruskal–Wallis test was used to compare medians between groups.

Multiple logistic regression analysis using the backward elimination method was performed to identify the factors associated with survival at discharge. Candidate variables for inclusion in the multiple logistic regression model were chosen from the univariate analysis; variables with *p* < 0.1 in the univariate analyses were included in the multivariate analysis, and collinearity was assessed before the multivariate analysis. Calibrations of the models were evaluated with the Hosmer–Lemeshow goodness-of-fit test. Statistical analyses were performed using the Statistical Package for the Social Sciences (SPSS) version 22.0 (IBM Corporation, Armonk, NY, USA).

## Results

### Baseline characteristics of the study population

During the study period (2012–2015), 5552 patients received ECMO support in Korea. ECMO support was given to 2472 patients in the participating 16 hospitals. We analyzed 487 (19.7%) patients who received ECMO specifically for respiratory failure. The annual number of ECMO cases at 16 institutions varied widely: eight centers had fewer than 20 cases per year and the other eight centers had more than 30 cases per year, with two of those centers having had more than 120 cases per year.

The patients’ median age was 58 years (range 45–66 years), and the median body mass index was 22.2 kg/m^2^ (range 20.6–23.2 kg/m^2^). Pre-ECMO mechanical ventilation was provided in 92.2% of patients and corticosteroid therapy was used in 16.8% of patients. Prone positioning was applied in 29.5% of patients and neuromuscular blockers were used in 45.4% of patients. The majority of patients were initially supported with veno-venous ECMO (88.1%), and the median duration of support was 8 days (interquartile range (IQR) 4, 18 days). Survival and weaning rates were 38.8% and 57.1%, respectively (Table 1).

**Table 1:** Baseline characteristics of patients supported with ECMO for respiratory failure

Variable	Total (*n* = 487)
Age (years)	58 (45, 66)
Male	321 (65.9)
Body mass index (kg/m^2^)	22.2 (20.6, 23.2)
APACHE II score	21 (15, 28)
SOFA score	8 (5, 12)
PRESERVE score	5 (4, 6)
RESP score	0 (−2, 2)
Etiology of ARF	
Viral pneumonia	47 (9.7)
Bacterial pneumonia	127 (26.1)
COPD and asthma	8 (1.6)
Trauma and burn	25 (5.1)
Asphyxia	13 (2.7)
Acute exacerbation of ILD	61 (12.5)
Chronic respiratory failure	24 (4.9)
ARDS	44 (9.0)
Airway obstruction	28 (5.7)
Other respiratory failure	110 (22.6)
Immunocompromised^a^	122 (25.2)
CNS dysfunction^b^	24 (5.0)
Vasopressor	301 (63.0)
Corticosteroid	82 (16.8)
Cardiac arrest	74 (15.2)
CRRT	83 (17.0)
Mechanical ventilation	449 (92.2)
Prone positioning	143 (29.5)
Pre-ECMO rescue therapy	
Nitric oxide	127 (26.2)
Bicarbonate infusion	53 (11.0)
Neuromuscular blocker	230 (45.4)
Vital signs	
MAP (mmHg)	70 (58, 84)
Heart rate (/min)	112 (95, 128)
Respiratory rate (/min)	22 (18, 28)
ECMO type	
Veno-venous	429 (88.1)
Veno-arterial	42 (8.6)
Veno-arteriovenous	14 (2.9)
Other	2 (0.4)
Arterial blood gases	
pH	7.28 (7.17, 7.38)
PaO_2_ (mmHg)	61 (51, 76)
PaCO_2_ (mmHg)	51 (39, 65)
HCO_3_^−^ (mEq/L)	23 (19, 29)
SaO_2_ (%)	88 (79, 93)
Ventilation parameters	
PaO_2_/FiO_2_	65 (53, 90)
FiO_2_	100 (90, 100)
PEEP (cmH_2_O)	10 (6, 12)
PIP (cmH_2_O)	28 (24, 32)
Tidal volume (ml/kg)	7 (6, 9)
Driving pressure (cmH_2_O)	18 (15, 24)
Minute ventilation (L/min)	9.6 (7.4, 12.4)
Interval MV–ECMO (days)	1 (0, 5)
ECMO duration (days)	8 (4, 18)
Hospital stay (days)	35 (18, 61)
Tracheostomy	199 (41.8)
Weaning rate	278 (57.1)
Survival rate	189 (38.8)

Values expressed as median (interquartile range) or *n* (%)

*ECMO* extracorporeal membrane oxygenation, *APACHE* Acute Physiology and Chronic Health Evaluation, *SOFA* Sequential Organ Failure Assessment, *PRESERVE* Predicting Death for Severe Acute Respiratory Distress Syndrome on Veno-venous ECMO, *RESP* Respiratory Extracorporeal Membrane Oxygenation Survival Prediction, *ARF* acute respiratory failure, *ARDS* acute respiratory distress syndrome, *COPD* chronic obstructive pulmonary disease, *ILD* interstitial lung disease, *CNS* central nervous system, *CRRT* continuous renal replacement therapy, *MAP* mean arterial pressure, *PaO*_*2*_ partial pressure of arterial oxygen, *PaCO*_*2*_ partial pressure of arterial carbon dioxide, *HCO*_*3*_^−^ bicarbonate, *SaO*_*2*_ oxygen saturation, *FiO*_*2*_ fraction of inspired oxygen, *PEEP* positive end-expiratory pressure, *PIP* peak inspiratory pressure, *MV* mechanical ventilation

^a^“Immunocompromised” included hematological malignancies, solid tumors, solid-organ transplantation, high-dose or long-term corticosteroid and/or immunosuppressant use, and human immunodeficiency virus infection

^b^“CNS dysfunction” included diagnoses of neurotrauma, stroke, encephalopathy, cerebral embolism, seizure, and epileptic syndrome

### Demographics, pre-ECMO parameters, and outcomes over time

**Fig. 1:**
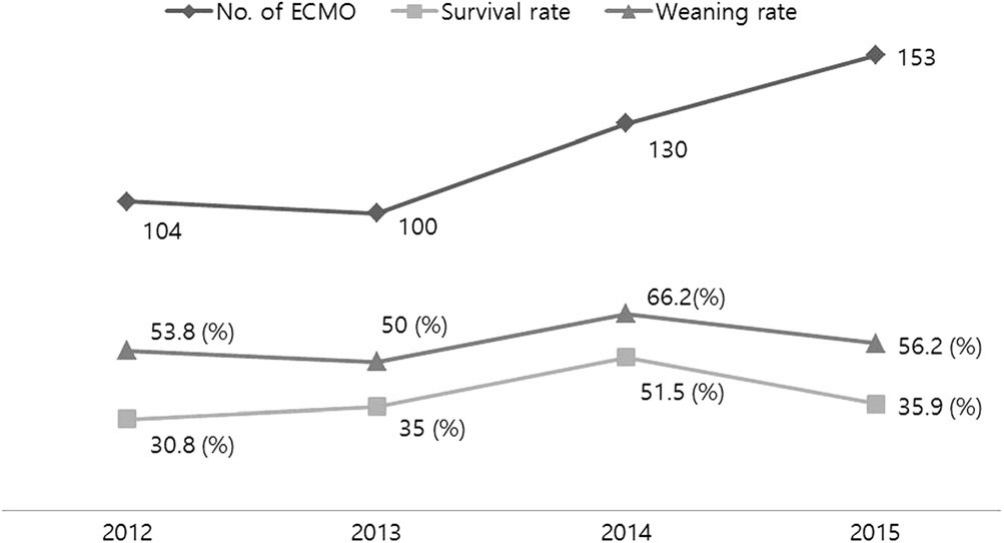
Number of ECMO procedures and weaning and survival rates of patients who received ECMO for acute respiratory failure. ECMO extracorporeal membrane oxygenation

The number of ECMO procedures for respiratory failure increased from 104 to 153 during the study period (Fig. 1). There were no significant differences in age, sex, APACHE II score, SOFA score, immunocompromised status, CNS dysfunction, cardiac arrest, CRRT, use of nitric oxide and bicarbonate infusion, PaO_2_/FiO_2_ ratio, ECMO duration, and duration of mechanical ventilation to ECMO initiation between groups. Use of prone positioning increased from 6.8% to 49.0% (*p* < 0.001) and the use of neuromuscular blockers also increased from 28.2% to 58.2% (*p* < 0.001; Table 2). Although the survival rate remained relatively low, it increased over time from 30.8% to 35.9% (*p* = 0.005; Table 3). Post-hoc analysis showed that the survival rate in 2014 was significantly higher than the rates in 2012 and 2015.

**Table 2:** Demographic features of survivors and nonsurvivors supported with ECMO for respiratory failure

Variable	2012 (*n* = 104)	2013 (*n* = 100)	2014 (*n* = 130)	2015 (*n* = 153)	*p* value
Age (years)	59 (49, 69)	60 (45, 68)	58 (43, 66)	57 (45, 63)	0.199
Male	69 (66.3)	71 (71.0)	93 (71.5)	88 (57.5)	0.050
Body mass index (kg/m^2^)	22.6 (20.4, 24.6)	22.9 (19.7, 25.0)	22.1 (21.0, 22.9)	22.0 (20.5, 22.9)	0.073
APACHE II score	21 (16, 27)	22 (16, 29)	21 (15, 30)	19 (14, 26)	0.162
SOFA score	8 (5, 12)	8 (5, 11)	8 (5, 12)	8 (6, 12)	0.842
PRESERVE score	5 (4, 7)	6 (4, 7)	5 (3, 6)	5 (3, 6)	0.245
RESP score	0 (−2, 2)	0 (−2, 2)	0 (−2, 2)	1(−1, 3)	0.497
Etiology of ARF					0.001
Viral pneumonia	7 (6.7)	8 (8.0)	11 (8.5)	21 (13.7)	
Bacterial pneumonia	33 (31.7)	16 (16.0)	37 (28.5)	41 (26.8)	
COPD and asthma	1 (1.0)	2 (2.0)	4 (3.1)	1 (0.7)	
Trauma and burn	1 (1.0)	4 (4.0)	10 (7.7)	10 (6.5)	
Asphyxia	0 (0.0)	3 (3.0)	8 (6.2)	2 (1.3)	
Acute exacerbation of ILD	8 (7.7)	17 (17.0)	13 (10.0)	23 (15.0)	
Chronic respiratory failure	11 (10.6)	4 (4.0)	6 (4.6)	3 (2.0)	
ARDS	13 (12.5)	14 (14.0)	7 (5.4)	10 (6.5)	
Airway obstruction	10 (9.6)	6 (6.0)	4 (3.1)	8 (5.2)	
Other respiratory failure	20 (19.2)	26 (26.0)	30 (23.1)	34 (22.2)	
Immunocompromised^a^	26 (25.2)	21 (21.4)	34 (26.2)	41 (26.8)	0.799
CNS dysfunction^b^	3 (2.9)	3 (3.1)	8 (6.2)	10 (6.5)	0.413
Vasopressor	48 (46.2)	59 (59.0)	96 (75.6)	98 (66.7)	< 0.001
Corticosteroid	22 (21.2)	21 (21.0)	23 (17.7)	16 (10.5)	0.068
Cardiac arrest	8 (7.7)	19 (19.0)	24 (18.5)	23 (15.0)	0.080
CRRT	20 (19.2)	19 (19.0)	20 (15.4)	24 (15.7)	0.783
Mechanical ventilation	94 (90.4)	83 (83.0)	125 (96.2)	147 (96.1)	< 0.001
Prone positioning	7 (6.8)	3 (3.1)	58 (44.6)	75 (49.0)	< 0.001
Pre-ECMO rescue therapy					
Nitric oxide	29 (28.2)	22 (22.4)	42 (32.3)	34 (22.2)	0.197
Bicarbonate infusion	11 (10.7)	12 (12.2)	14 (10.8)	16 (10.5)	0.975
Neuromuscular blocker	29 (28.2)	32 (32.7)	80 (61.5)	89 (58.2)	< 0.001

Values expressed as median (interquartile range), mean ± standard deviation, or *n* (%)

*ECMO* extracorporeal membrane oxygenation, *APACHE* Acute Physiology and Chronic Health Evaluation, *SOFA* Sequential Organ Failure Assessment, *PRESERVE* Predicting Death for Severe Acute Respiratory Distress Syndrome on Veno-venous ECMO, *RESP* Respiratory Extracorporeal Membrane Oxygenation Survival Prediction, *ARF* acute respiratory failure, *COPD* chronic obstructive pulmonary disease, *ILD* interstitial lung disease, *ARDS* acute respiratory distress syndrome, *CNS* central nervous system, *CRRT* continuous renal replacement therapy

^a^“Immunocompromised” included hematological malignancies, solid tumors, solid-organ transplantation, high-dose or long-term corticosteroid and/or immunosuppressant use, and human immunodeficiency virus infection

^b^“CNS dysfunction” included diagnoses of neurotrauma, stroke, encephalopathy, cerebral embolism, seizure, and epileptic syndrome

**Table 3:** Pre-ECMO parameters of patients supported with ECMO for respiratory failure

Variable	2012 (*n* = 104)	2013 (*n* = 100)	2014 (*n* = 130)	2015 (*n* = 153)	*p* value
Vital signs					
MAP (mmHg)	74 (62, 89)	72 (59, 86)	63 (56, 72)	70 (57, 84)	0.001
Heart rate (/min)	112 (98, 125)	116 (101, 131)	107 (94, 125)	112 (94, 129)	0.462
Respiratory rate (/min)	26 (20, 30)	24 (20, 30)	20 (16, 26)	20 (16, 26)	< 0.001
ECMO type					0.003
Veno-venous	96 (92.3)	95 (95.0)	113 (86.9)	125 (81.7)	
Veno-arterial	4 (3.8)	2 (2.0)	11 (8.5)	25 (16.3)	
Veno-arteriovenous	3 (2.9)	2 (2.0)	6 (4.6)	3 (2.0)	
Other	1 (1.0)	1 (1.0)	0 (0.0)	0 (0.0)	
Arterial blood gases					
pH	7.31 (7.17, 7.43)	7.25 (7.17, 7.36)	7.26 (7.15, 7.37)	7.29 (7.18, 7.38)	0.081
PaO_2_ (mmHg)	60 (52, 74)	66 (56, 79)	62 (50, 75)	61 (46, 76)	0.211
PaCO_2_ (mmHg)	52 (40, 62)	56 (41, 72)	51 (39, 71)	47 (36, 59)	0.013
HCO_3_^−^ (mEq/L)	24.3 (21.1, 31.0)	24.1 (19.9, 29.5)	22.4 (18.1, 27.9)	22.0 (18.2, 25.5)	0.003
SaO_2_ (%)	88 (83, 92)	89 (85, 94)	88 (79, 93)	87 (75, 93)	0.243
Ventilation parameters					
PaO_2_/FiO_2_	62 (53, 80)	72 (59, 96)	65 (53, 90)	65 (48, 97)	0.131
FiO_2_	100 (100, 100)	100 (90, 100)	100 (80, 100)	100 (80, 100)	0.069
PEEP (cmH_2_O)	10 (6, 12)	8 (5, 12)	10 (6, 10)	10 (7, 12)	0.119
PIP (cmH_2_O)	28 (24, 33)	30 (25, 34)	28 (23, 33)	28 (24, 31)	0.382
Tidal volume (ml/kg)	389 (298, 575)	420 (321, 513)	444 (340, 600)	428 (299, 518)	0.255
Driving pressure (cmH_2_O)	18 (14, 24)	20 (16, 25)	18 (15, 23)	18 (15, 21)	0.077
Minute ventilation (L/min)	10.9 (7.8, 14.6)	9.6 (7.7, 12.7)	9.5 (7.2, 12.2)	9.3 (6.8, 12.0)	0.035
Interval MV–ECMO (days)	2 (0, 7)	1 (0, 5)	1 (0, 5)	2 (0, 5)	0.090
ECMO duration (days)	7 (4, 14)	8 (5, 22)	8 (3, 13)	7 (4, 24)	0.305
Hospital stay (days)	32 (17, 47)	34 (19, 65)	39 (18, 73)	34 (17, 60)	0.318
Tracheostomy	32 (30.8)	38 (38.0)	61 (48.8)	68 (46.3)	0.023
Weaning rate	56 (53.8)	50 (50.0)	86 (66.2)	86 (56.2)	0.075
Survival rate	32 (30.8)	35 (35.0)	67 (51.5)	55 (35.9)	0.005

Values expressed as mean ± standard deviation, or *n* (%)

*ECMO* extracorporeal membrane oxygenation, *MAP* mean arterial pressure, *PaO*_*2*_ partial pressure of oxygen, *PaCO*_*2*_ partial pressure of carbon dioxide, *HCO*_*3*_^−^ bicarbonate, *SaO*_*2*_ oxygen saturation, *FiO*_*2*_ fraction of inspired oxygen, *PEEP* positive end-expiratory pressure, *PIP* peak inspiratory pressure, *MV* mechanical ventilation

### Factors associated with mortality in patients supported with ECMO

Multiple regression analysis was performed using age, sex, year, APACHE II score, SOFA score, immunocompromised status, CNS dysfunction, corticosteroid, CRRT, prone positioning, nitric oxide, neuromuscular blocker, PaCO_2_, peak inspiratory pressure, driving pressure, and ECMO duration. Old age (OR 1.038 (95% CI 1.022, 1.054)), use of corticosteroid (OR 2.251 (95% CI 1.153, 4.397)), CRRT (OR 2.196 (95% CI 1.135, 4.247)), driving pressure (OR 1.072 (95% CI 1.031, 1.114)), and prolonged ECMO duration (OR 1.020 (95% CI 1.003, 1.038)) were associated with increased odds of mortality (Table 4).

**Table 4:** Univariate and multivariate analyses for mortality of ECMO

Variable	OR (95% CI)	*p* value	OR (95% CI)	*p* value
Age (years)	1.043 (1.029, 1.056)	< 0.001	1.038 (1.022, 1.054)	< 0.001
Male	0.720 (0.487, 1.064)	0.099		
Year	0.645 (0.444, 0.939)	0.022		
Body mass index (kg/m^2^)	0.982 (0.927, 1.040)	0.527		
APACHE II score	1.032 (1.010, 1.054)	0.004		
SOFA score	1.020 (0.978. 1.065)	0.358		
Immunocompromised	1.335 (0.868, 2.054)	0.188		
CNS dysfunction	1.274 (0.534, 3.038)	0.585		
Vasopressor	1.254 (0.857, 1.836)	0.243		
Corticosteroid	1.914 (1.130, 3.242)	0.016	2.251 (1.153, 4.397)	0.018
Cardiac arrest	1.050 (0.630, 1.747)	0.852		
CRRT	2.102 (1.233, 3.581)	0.006	2.196 (1.135, 4.247)	0.019
Prone positioning	1.054 (0.705, 1.575)	0.798		
Nitric oxide	1.853 (1.194, 2.875)	0.006		
Bicarbonate infusion	1.521 (0.820, 2.820)	0.183		
Neuromuscular blocker	1.186 (0.821, 1.711)	0.363		
VV ECMO mode	0.810 (0.456, 1.439)	0.472		
pH	0.800 (0.244, 2.618)	0.712		
PaO_2_ (mmHg)	0.998 (0.993, 1.003)	0.433		
PaCO_2_ (mmHg)	1.007 (0.999, 1.015)	0.083		
PaO_2_/FiO_2_	0.999 (0.996, 1.002)	0.447		
PEEP (cmH_2_O)	0.968 (0.918, 1.020)	0.219		
PIP (cmH_2_O)	1.070 (1.034, 1.107)	< 0.001		
Tidal volume (ml/kg)	0.999 (0.998, 1.000)	0.175		
Driving pressure (cmH_2_O)	1.078 (1.039, 1.118)	< 0.001	1.072 (1.031, 1.114)	< 0.001
Minute ventilation (L/min)	1.022 (0.969, 1.077)	0.428		
Interval MV–ECMO (days)	1.024 (0.998, 1.050)	0.068		
ECMO duration (days)	1.016 (1.003, 1.029)	0.017	1.020 (1.003, 1.038)	0.021

*ECMO* extracorporeal membrane oxygenation, *OR* odds ratio, *CI* confidence interval, *APACHE* Acute Physiology and Chronic Health Evaluation, *SOFA* Sequential Organ Failure Assessment, *CNS* central nervous system, *CRRT* continuous renal replacement therapy. *VV* veno-venous, *PaO*_*2*_ partial pressure of oxygen, *PaCO*_*2*_ partial pressure of carbon dioxide, *FiO*_*2*_ fraction of inspired oxygen, *PEEP* positive end-expiratory pressure, *PIP* peak inspiratory pressure, *MV* mechanical ventilation

**Fig. 2:**
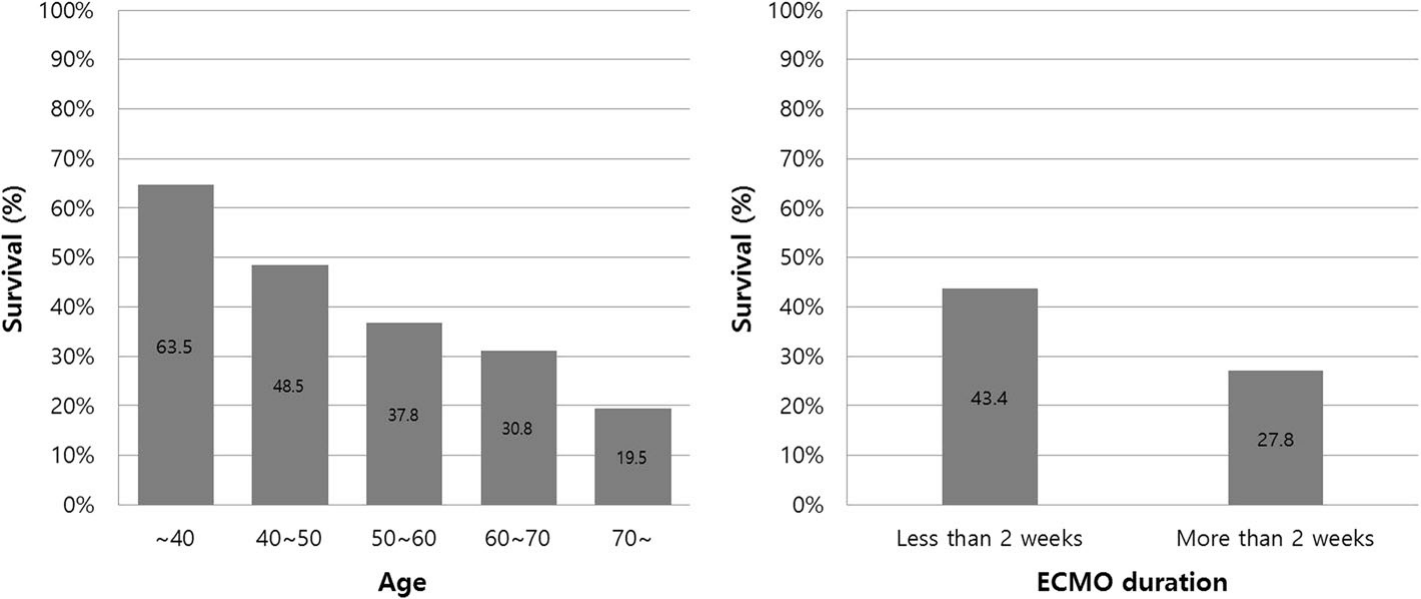
Survival rates of ECMO patients according to age (years) (*p* < 0.001) and ECMO duration (*p* = 0.001). ECMO extracorporeal membrane oxygenation

The median age was older in the nonsurvivors (61 years; IQR 52, 69 years) than in survivors (51 years; IQR 37, 62 years) (*p* < 0.001). The survival rate decreased with age, with patients older than 60 years having a survival rate of 30.8% (Fig. 2). ECMO duration was significantly longer in the nonsurvivors (9 days; interquartile range (IQR) 4, 22 days) than in survivors (7 days; IQR 3, 13 days) (*p* = 0.002). Compared with the survival rate within 2 weeks of ECMO support, the overall survival rate after 2 weeks of ECMO support showed a significant decrease from 43.4% to 27.8% (*p* = 0.001).

## Discussion

This multicenter study was conducted to evaluate the change in survival rates of patients who received ECMO support for acute respiratory failure in Korea. Utilization of ECMO for respiratory failure increased over time, and the survival rate was improved with increasing use of adjunctive management. Also, patient age and the duration of ECMO were significantly associated with survival.

A notable change during the study period was that the administration of neuromuscular blockades and use of prone positioning before ECMO had significantly increased from 28.2% to 58.2% and from 6.8% to 49.0%, respectively. Papazian et al. [13] reported that early use of neuromuscular blockades in patients with severe ARDS may improve survival. In the ELSO registry-based RESP study, neuromuscular blockade agents before ECMO were independently associated with hospital survival [12]. In addition, in patients with severe ARDS, early application of prolonged prone positioning was significantly associated with improved survival [14]. Schmidt et al. [15] demonstrated that use of prone positioning before ECMO was also associated with survival. These results are in accordance with those in a recent systematic review and meta-analysis [16]. Moreover, for patients with severe ARDS, prone positioning before and during ECMO may be helpful for weaning from ECMO [17, 18]. Another distinctive finding was the change in pre-ECMO ventilator parameters. In recent years, the driving pressure was lower and minute ventilation was decreased. Therefore, improvement in hospital survival of ECMO-supported patients with respiratory failure might be the result of increasing experience with ECMO over time, including evolving adjuvant therapies and improved management of mechanical ventilation.

The results of this study showed that the number of ECMOs carried out for respiratory failure increased from 104 to 153 from 2012 to 2015, and that the in-hospital survival rate increased from 30.8% to 35.9% during the same period. The overall survival rate of 39% in ECMO-supported respiratory failure patients in Korea is lower than the reported rate of 58% in the ELSO registry [7]. Meanwhile, an ECMO epidemiologic study performed in Germany reported that from 2012 to 2014 the in-hospital survival had steadily increased and the rate of survival was approximately 40%, which is similar to our findings [8]. In addition, Sauer et al. [9] reported that in the United States the survival rate of the patients who received ECMO was approximately 40%. In the German study, approximately 80% of patients were older than 40 years and increasing numbers of older patients had received ECMO. In the US study, the mean age of the patients who received ECMO was 50 years, which is higher than that of the patients included in the ELSO registry. Taken together, the discrepancies in demographics between the patients of ECMO centers not included in the ELSO and those in the ELSO registry may explain the difference in survival rates. Also, another explanation for the relatively low survival rate of Korean ECMO patients could be the infrequent use of prone positioning. The use of prone positioning and use of neuromuscular blockers were low compared with those in the EOLIA trial [6], in which prone positioning was applied in 90% of patients in the conventional ventilator support group, who showed a 54% survival rate. The relatively low survival rate in Korean ECMO patients may be due to excessive use of ECMO in patients who may have shown good response to prone positioning. Accordingly, the use of prone positioning is gradually increasing in Korea.

Another interesting finding of our study was that the survival rate was associated with the ECMO duration. The survival rate of patients who required prolonged ECMO (longer than 14 days) was significantly lower than that of patients who had shorter ECMO duration (28% vs 43%, respectively, *p* = 0.001). Recently, Posluszny et al. [19] reported that ECMO duration was inversely correlated with the survival rate in ECMO-supported patients with respiratory failure; the survival rate in patients who had longer ECMO duration was 10% lower than that in those with shorter ECMO duration. Nonetheless, the investigators suggested that prolonged ECMO was not futile because there was a significant improvement in survival from 37% to 49% in recent years. On the other hand, the aforementioned German epidemiologic study reported that prolonged ECMO was associated with poorer outcome; that the survival rate rapidly declined to 20% within 10 days after ECMO initiation [8]. Therefore, further studies are needed to provide a more solid association between ECMO duration and the survival rate.

Our study has several limitations. This study was retrospective and had a relatively short study period. Because not all patients treated with ECMO for respiratory failure in Korea were included, selection bias is possible. In addition, long-term outcomes and quality of life could not be assessed, which warrants an extended observation period of our study populations or further epidemiologic studies. Despite such limitations, our current multicenter study, which is not based on the ELSO registry, provides information on the change in the survival rate of ECMO patients with respiratory failure and the factors associated with survival, and adds to the understanding of survival in patients who receive ECMO due to respiratory failure.

## Conclusions

This multicenter study performed in Korea showed that utilization of ECMO for respiratory failure had increased over time, and that the survival rates of ECMO-supported respiratory failure patients had improved with increasing utilization of adjunctive management. Patient age and duration of ECMO were significantly associated with survival at discharge.

## Abbreviations

APACHE: Acute Physiology and Chronic Health Evaluation
ARDS: Acute respiratory distress syndrome
CNS: Central nervous system
CRRT: Continuous renal replacement therapy
ECMO: Extracorporeal membrane oxygenation
ELSO: Extracorporeal Life Support Organization
ICU: Intensive care unit
IQR: Interquartile range
OR: Odds ratio
SOFA: Sequential Organ Failure Assessment
